# High-Strength and Rapidly Degradable Nanocomposite Yarns from Recycled Waste Poly(glycolic acid) (PGA)

**DOI:** 10.3390/polym17010100

**Published:** 2025-01-02

**Authors:** Ben Liu, Shixiao Wang, Hanling Guo, Huibo Yin, Yuqiu Song, Min Gong, Liang Zhang, Xiang Lin, Dongrui Wang

**Affiliations:** 1 State Key Laboratory of Shale Oil and Gas Enrichment Mechanisms and Effective Development, No. 197 Baisha Road, Shahe Town, Beijing 102206, China; liubenblue1201@sina.com (B.L.); yinhb.sripe@sinopec.com (H.Y.); 2 Research and Development Center of Measurement and Control Technology and Equipment, SINOPEC Research Institute of Petroleum Engineering Co., Ltd., No. 197 Baisha Road, Shahe Town, Beijing 102206, China; 3 School of Chemistry and Biological Engineering, University of Science and Technology Beijing, No. 30 Xueyuan Road, Haidian District, Beijing 100083, China; d202410502@xs.ustb.edu.cn (S.W.); d202210465@xs.ustb.edu.cn (H.G.); syqiu1008@163.com (Y.S.); gongmin@ustb.edu.cn (M.G.); zhangliang@ustb.edu.cn (L.Z.); linxiang@ustb.edu.cn (X.L.)

**Keywords:** poly(glycolic acid), nanocomposite yarn, electrospinning, graphene oxide nanosheets, carbon nanotubes

## Abstract

Poly(glycolic acid) (PGA) is a rapidly degradable polymer mainly used in medical applications, attributed to its relatively high cost. Reducing its price will boost its utilization in a wider range of application fields, such as gas barriers and shale gas extraction. This article presents a strategy that utilizes recycled PGA as a raw material alongside typical carbon nanomaterials, such as graphene oxide nanosheets (GO) and carbon nanotubes (CNTs), to produce low-cost, fully degradable yarns via electrospinning and twisting techniques. The results demonstrate that the tensile strength of the PGA/GO composite yarn increased to 21.36 MPa, and the elastic modulus attained a value of 259.51 MPa with a 3 wt% of GO loading. The addition of an appropriate amount of GO enhances the tensile resistance of the composite yarns to a certain extent. However, excessive application of GO and CNTs can lead to surface defects in the nanofibers, reducing their mechanical properties. Moreover, the integration of both materials could inhibit the degradation process of PGA to some extent, thereby partially addressing the issue of excessive degradation rates associated with the relatively low molecular weight of recycled PGA.

## 1. Introduction

The development of environmentally friendly and biodegradable polymers and associated products has garnered increasing interest in the past few decades, owing to the world’s escalating concern regarding environmental protection and accumulating plastic waste pollution. Polyglycolic acid (PGA), as a representative synthetic aliphatic polyester, has long been known for its simple chemical structure and excellent biocompatibility, mechanical strength, and biodegradability [1]. The global PGA market is projected to exceed USD 9 billion in 2024, according to the market research results. Nonetheless, its application domain is primarily confined to the medical industry. The most common uses of PGA in medical applications include absorbable sutures and other advanced materials such as biodegradable bone grafts, dental materials, scaffolds for tissue engineering, and drug delivery systems [2,3,4,5,6]. A major reason leading to this situation is the high cost of PGA, due to the significant propensity for depolymerization during polymerization, which inhibits the synthesis of high-molecular-weight PGA [7,8].

The advancement of affordable and high-performance PGA products is crucial for expanding their range of applications. To lower the cost of PGA products and broaden their applications, including in the packaging sector and shale gas extraction, extensive efforts are being made in polymerization. This involves employing various copolymerization techniques to develop PGA-containing copolymers [8,9,10]. The cost of the synthesized copolymers can be minimized while simultaneously allowing for regulations to the chain structures and physical properties, such as mechanical strength and degradation behavior. Furthermore, there are several studies that examine processing and molding techniques, particularly through blending, focusing on the development of multiphasic polymer blends by compositing PGA with other biodegradable polymers [11,12,13]. Additionally, several investigations have utilized the electrospinning method for manufacturing PGA or PGA-based composite nanofibers aimed at applications like tissue engineering scaffolds and air filtration membranes [14,15,16,17,18,19,20]. It is crucial to note that PGA is insoluble in most organic solvents except for 1,1,1,3,3,3 hexafluoro-2-propanol (HFIP), and that the molecular weight of PGA should not exceed a certain limit. Consequently, the mechanical characteristics of PGA nanofibers generated via electrospinning are comparatively inadequate, making them inappropriate for specific applications that require elevated mechanical strength. Over the past few years, carbon nanomaterials such as carbon nanotubes (CNTs) and graphene nanosheets have been extensively employed in co-spinning with polymers to create nanocomposite fibers that exhibit enhanced mechanical performance. However, to date, studies on the electrospinning of carbon nanomaterial-filled PGA nanocomposites are quite limited. The majority of studies concentrate on employing PGA-containing copolymers, such as poly(lactic-co-glycolic acid) (PLGA) [21,22,23], to create composite nanofibers reinforced with CNTs.

This article presents a strategy that employs waste PGA as the raw material for compositing with conventional carbon nanomaterials, including graphene oxide nanosheets (GO) and CNTs, to create low-cost, fully degradable yarns through electrospinning and twisting techniques. The pricing of recycled PGA is significantly lower; however, their decreased average molecular weight makes their products almost ineffective. It is found that the PGA yarns fabricated from the electrospun nanofiber films displayed adequate mechanical properties. By incorporating suitable amount of GO into the PGA nanofibers, their tensile strength can further be enhanced. However, the addition of excessive amounts of GO and CNTs led to a deterioration in tensile strength. Meanwhile, the degradation of the nanocomposite yarns in water is slightly decreased by the introduction of GO and CNTs. The resultant cost-effective and fully degradable nanocomposite yarns may find practical applications in some new areas like shale gas extraction.

## 2. Experimental Section

Materials: recycled PGA powders with a melting index of 40–50 g/10 min were obtained from Shenzhen Polymtek Biomaterial (Shenzhen, China). 1,1,1,3,3,3 hexafluoro-2-propanol (HFIP, >99.5%) was purchased from Shanghai Aladdin Chemicals (Shanghai, China). GO nanosheets and multiwalled carbon nanotubes were obtained from XF Nano (Nanjing, China). The morphologies of the GO and CNT samples are shown in Figure S1 and Figure S2, respectively. All other chemicals were commercially available products and used as received.

Fabrication of nanofibers and yarns: firstly, stock solutions for electrospinning were prepared by dissolving certain amount of PGA into HFIP under stirring at 50 °C overnight. In preparing the composite spinning solutions, the necessary amount of GO or CNTs was carefully measured in advance and then dispersed in HFIP through ultrasonication. The dispersion was subsequently mixed with the PGA solution to formulate a homogeneous spinning solution. An optimal concentration of PGA in the dispersion was confirmed to be 15 wt%, with GO addition in the range of 0–5 wt% or CNT addition of 0–3 wt%. The obtained spinning solution was then loaded into a syringe with a stainless-steel needle (22G) at its end, which was connected to a syringe pump, a direct current power supply, and a grounded collector covered with aluminum foil. The feeding rate of the solution was controlled to be 1.0 mL/h, and the applied voltage was set to be 10–20 kV with a distance between the needle tip and the collector of 25 cm. The electrospinning was performed under room temperature with a relative humidity of 25–30%. After drying under vacuum at 60 °C for 12 h, the obtained nanofiber films were then carefully peeled off the aluminum foil and cut into strips. PGA, PGA/GO, and PGA/CNT yarns were finally prepared by twisting the nanofiber strips into yarns with desired diameters. Typically, strips of the nanofiber films with a size of 8 × 1 cm^2^ were twisted into yarns with a nominal diameter of ca. 0.5 mm at a twisting rate of 60 rpm (1 twist/s).

Characterizations: the morphology of the nanofibers was observed by using scanning electron microscopy (SEM, SU8010, Hitachi, Tokyo, Japan) under an acceleration voltage of 10 kV. The samples were sputtered with gold nanoparticles prior to observation. The tensile tests of the yarns were performed by using a mechanical testing system (ESM303, Mark-10, Copiague, NY, USA) equipped with a sensor of 500 N. Yarns with a nominal diameter of ca. 0.5 mm and length of 50 mm were tested. The deformation rate during tensile tests was set as 13 mm/min. All the tensile measurements were performed under room temperature and ambient conditions. Averaged results were reported for each sample after testing at least five specimens. The degradation of the nanocomposite yarns was performed by immersing the samples in distilled water at 60 °C for determined time intervals. Then, the sample was removed from the water, freeze dried, and weighed using an electronic balance under room temperature.

## 3. Results and Discussion

**Figure 1:**
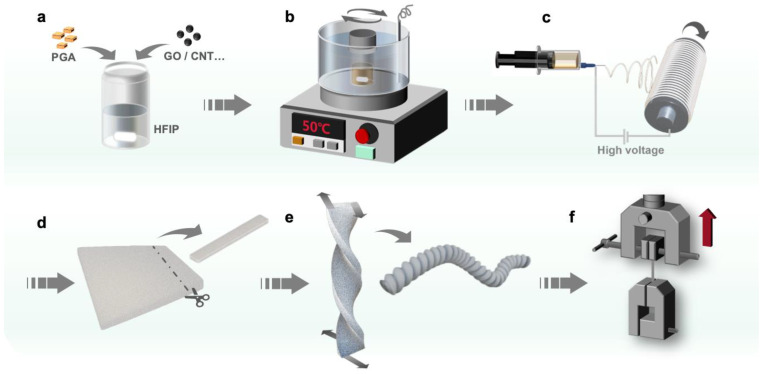
Schematic of fabrication of PGA/GO and PGA/CNT nanocomposite yarns. (**a**) Weighing the raw materials; (**b**) preparation of solutions for electrospinning via heating and stirring; (**c**) electrospinning of the nanofibers; (**d**) cutting the nanofiber films into strips; (**e**) twisting the strips into nanocomposite yarns; (**f**) evaluating the tensile properties of the yarns.

The fabrication of nanocomposite yarns by incorporating recycled PGA with GO nanosheets or CNTs is illustrated in Figure 1. Firstly, recycled PGA and GO or CNTs, serving as fillers, are mixed in HFIP under heating and stirring to create a homogeneous spinning solution. Subsequently, the spinning solution is processed into a nanofiber film via the electrospinning technique. Following this, the nanofiber film is cut into strips, twisted along its length, and transformed into yarn. The PGA powders used herein are a recycled material originally intended for the production of biodegradable medical products. Following long-term storage and some degradation of the macromolecular chains, the average molecular weight has decreased, leading to solubility in HFIP and making it appropriate for electrospinning. Nonetheless, because of the low molecular weight of the recycled PGA, the mechanical strength is significantly diminished in comparison to typical PGA samples with high molecular weight.

**Figure 2:**
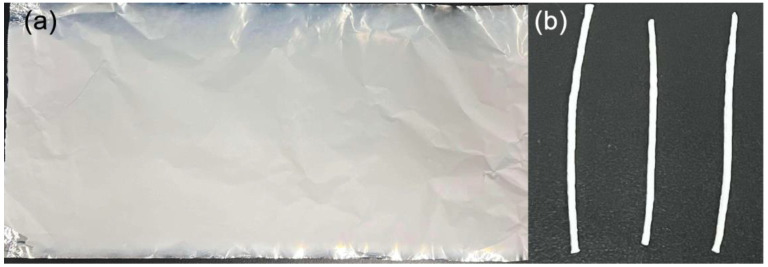
(**a**) Optical image of an electrospun PGA nanofiber film deposited on Al foil; (**b**) typical PGA yarns prepared through twisting the electrospun PGA nanofiber film.

Figure 2 presents the typical optical images of the obtained nanofiber films and yarns. A roller was utilized as the receiving device for nanofibers during the electrospinning process in this study. In the course of the rolling reception process, the charged jets were positioned on the aluminum foil on the roller’s surface in a specific orientation, leading to a defined alignment of the nanofibers. This alignment could potentially result in anisotropic mechanical properties within the nanofiber films. Table S1 (in the Supporting Information) gives the tensile properties of the yarns twisted from the electrospun nanofiber film made of pure PGA, with both parallel and perpendicular orientations to the rolling direction. The results reveal that the tensile strength and elastic modulus of yarns measured in the parallel direction exceed those measured in the perpendicular direction, whereas the elongation at break is reduced. To achieve yarn with enhanced strength, the following investigations in this study have utilized nanofiber films aligned with the rolling direction of the roller as the starting material for the nanocomposite yarn fabrication.

**Figure 3:**
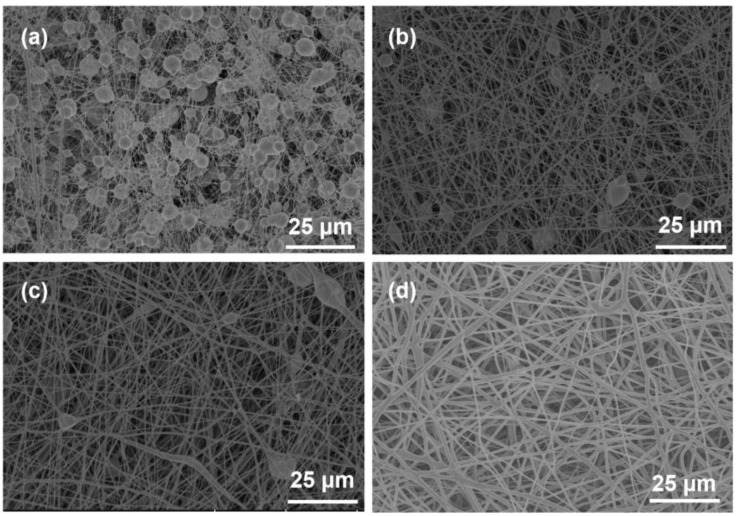
Typical SEM images of electrospun nanofiber films from PGA solutions with varying concentrations. (**a**–**d**) correspond to solutions with concentrations of 8 wt%, 10 wt%, 12 wt%, and 15 wt%, respectively.

**Figure 4:**
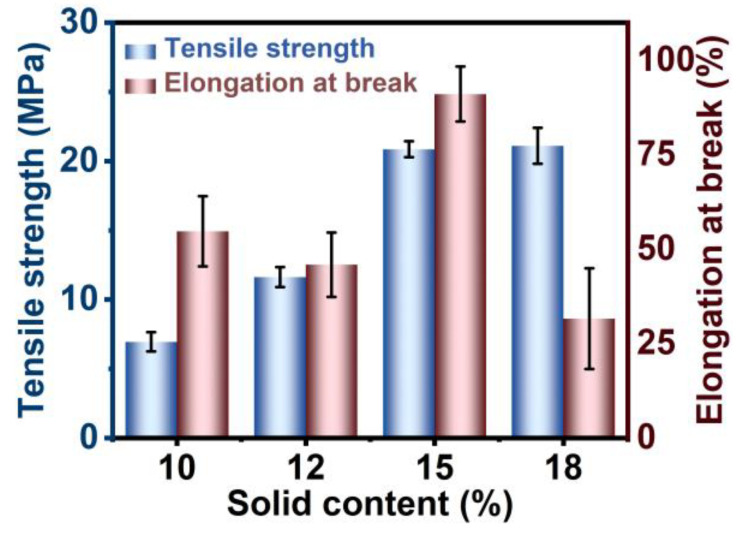
Mechanical properties of PGA yarns from nanofiber films using the spinning solutions with various solid contents.

The processing parameters of electrospinning significantly influence the morphology and physical properties of the nanofibers [24]. The influence of the voltage and the concentration of the spinning solution on the morphology and mechanical characteristics of the resultant nanofibers is investigated in detail. As shown in Figure S3, the resultant PGA yarns show better tensile strength under higher electrospinning voltage within the range of 10–20 kV. In addition, PGA solutions with concentrations of 8 wt%, 10 wt%, 12 wt%, 15 wt%, and 18 wt% were compared for the electrospinning. Figure 3 displays the SEM images of the nanofiber morphologies using the solution with varying solid contents, whereas Figure 4 illustrates the tensile strength and elongation at break of the resultant spun films. Figure 3a illustrates that at a solid content of 8 wt%, the majority of the solution is fragmented into small beads due to the influence of electrostatic force. The presence of these beads interrupts the continuity of the fibers, thereby inhibiting the development of high-quality nanofiber films. Figure 3b–d indicate that with an increase in solid content, the beads within the fiber film are progressively elongated into fibers due to electrostatic forces. Concurrently, the quantity and dimensions of droplets diminish. As shown in Figure 3d, when the solid content of the solution increases to 15 wt%, there are no discernible droplets on the surface of the nanofiber film, indicating a relatively uniform non-woven structure in which PGA nanofibers are loosely and randomly packed together. Figure 4 illustrates that an increase in the solid content of the spinning solution correlates with an increase in the tensile strength of the PGA nanofiber yarns. Low solid content results in reduced tensile strength, making it an undesirable option. At solid contents of 15 wt% and 18 wt%, the differences in tensile strength are small; however, the elongation at break is greater at 15 wt% solid content, indicating that the yarn exhibits superior tensile strength at this concentration. These results indicate that a solid content of 15 wt% is the optimal concentration for the spinning solution.

**Figure 5:**
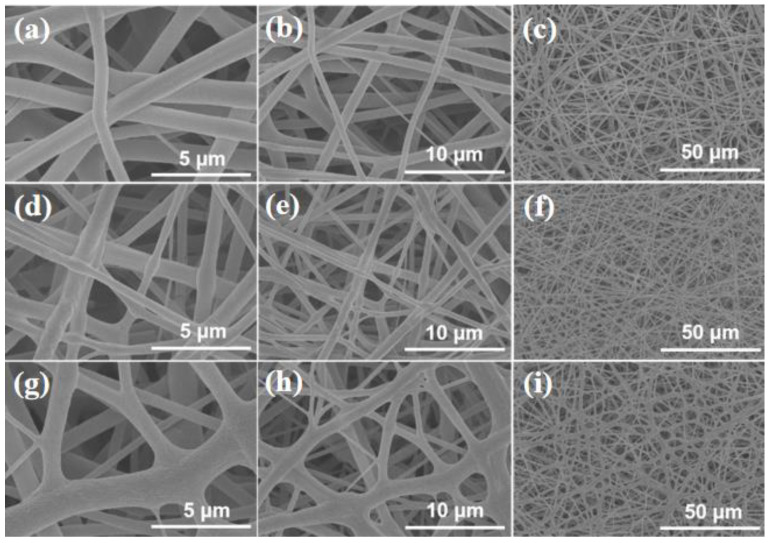
Typical SEM images of electrospun nanofibers. (**a**–**c**) pure PGA; (**d**–**f**) PGA:GO = 99:1 in wt:wt; (**g**–**i**) PGA:GO = 97:3 in wt:wt.

After the determination of the electrospinning conditions for PGA, certain amounts of GO nanosheets and CNTs were incorporated into the spinning solution to produce nanocomposite fiber films. Figure 5 displays SEM images of the resultant PGA/GO nanocomposite fiber films with varying mass fractions of GO nanosheets. It is shown that the fiber structures of the PGA/GO nanocomposite films are relatively uniform with no defects or GO aggregates. This implies that the distribution of GO in PGA nanofibers is uniform. The average fiber diameter is measured to be approximately 700 nm with a GO loading of 1 wt% and increases to 800–1000 nm at a GO concentration of 3 wt%. Comparing the surfaces of nanofibers made from pure PGA and those with 1 wt% of GO loading reveals that the latter’s surfaces become rather rough, indicating that the incorporation of GO may elevate the likelihood of defects in the surface of nanofibers. With the further increase in the amount of GO loading, the diameter of the fiber expands, resulting in widely distributed diameters and adhesion among the fibers. Figure S4 presents the SEM images of PGA/CNT nanocomposite fiber films with various mass fractions of CNTs. Similar to PGA/GO nanocomposite films, the CNT-doped fiber films also exhibit a relatively compact structure, resulting in fibers that are uniform in appearance. The surface of nanofibers doped with CNTs exhibit fewer defects than that of nanofibers composited with GO. As the added amount of CNTs increases, the diameter of the nanofibers also increases. The fiber diameter ranges from 600 to 700 nm with the addition of 1 wt% of CNTs and from 700 to 900 nm with the addition of 3 wt%. Furthermore, in comparison to the previous case, the diameter of the nanofibers with a 3 wt% of CNT addition exhibits greater variability.

**Figure 6:**
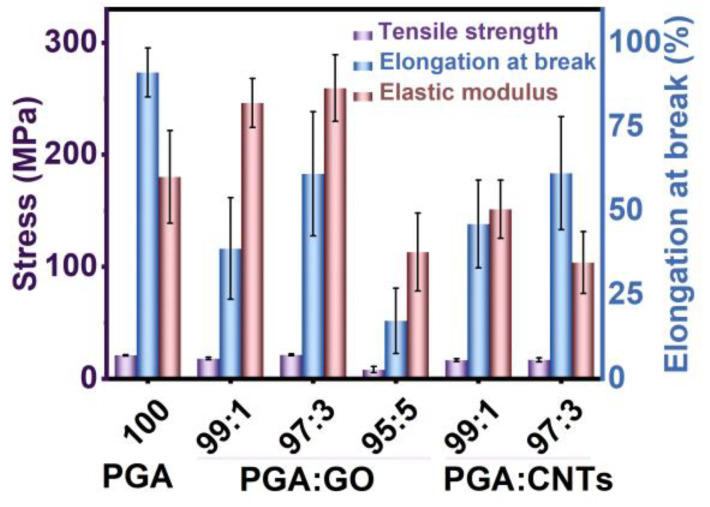
Tensile strength, elastic modulus, and elongation at break of PGA yarns and PGA-based nanocomposite yarns fabricated from the electrospun nanofiber films.

Figure 6 displays the mechanical properties of PGA/GO and PGA/CNT nanocomposite yarns, with the associated information listed in Table 1. The addition of GO at 3 wt% results in a largely reduced maximum elongation of the composite yarn, which is about 60.98%. This suggests that the incorporation of GO increases the brittleness of the PGA/GO yarn. However, the incorporation of GO resulted in an increase in the tensile strength of the yarn to 21.36 MPa and an elastic modulus of 259.51 MPa, thereby enhancing the material’s rigidity and tensile load capacity. With further increasing of the GO content, the tensile strength of PGA/GO composite yarns exhibits a declining trend, while both the elongation at break and elastic modulus consistently decrease. The excessive addition of GO led to an increased number of material defects, which resulted in a decline in mechanical properties. The SEM analysis indicates that the incorporation of GO leads to a continuous increase in the diameter of the nanofibers, a reduction in the spacing between the fibers, and the observation of adhesion in certain fiber films. The addition of a suitable amount of GO enhances the interaction between fibers, which in turn increases their strength and elastic modulus. Excessive addition of GO can cause substantial structural defects in the fibers, leading to a marked decrease in the mechanical properties of the resultant yarn.

For PGA/CNT nanocomposite yarns, the introduction of CNTs at 1 wt% or 3 wt% results in a reduction in the mechanical properties compared to pure PGA yarns. The tensile strength of the PGA/CNT nanocomposite yarn with a 3 wt% addition decreased to 16.75 MPa, while the elongation at break reduced to 46.06%, and the elastic modulus fell to 151.28 MPa. The incorporation of CNTs results in increased brittleness of the composite nanofibers. The mechanical characteristics of nanocomposite PGA yarns filled with GO and CNTs are noticeably different. This may stem from the enhancing mechanisms influenced by the dimensional factor and size of the nanofillers, which will be examined in more detail in future studies. In terms of enhancing the mechanical strength of PGA composite materials, the incorporation of two-dimensional GO nanosheets proves to be more effective than the addition of one-dimensional CNTs.

**Figure 7:**
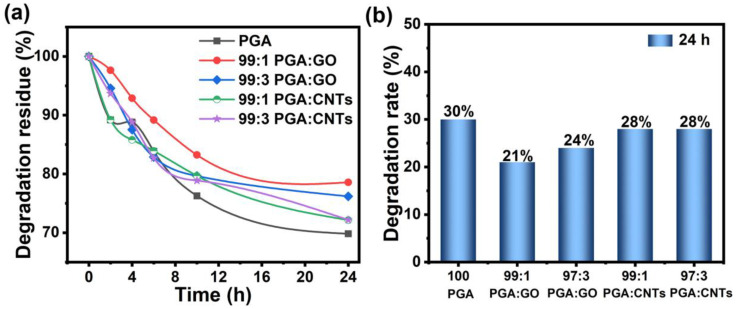
Degradation behavior of the PGA and PGA-based nanocomposite yarns in water at 60 °C. (**a**) Residue rate of the yarns over different degradation time intervals; (**b**) calculated degradation rate of various yarns after 24 h.

The degradation characteristics of the resultant composite yarns were also examined. Figure 7 illustrates the degradation efficiency of several composite yarns in distilled water at 60 °C. Figure 7a displays that the degrading process of PGA/GO and PGA/CNT composite yarns parallels that of pure PGA yarns, exhibiting a high degradation rate during the initial 10 h, succeeded by a decelerated degradation rate. The degradation experiments in this work were performed in a distilled water environment, resulting in degradation primarily attributed to the hydrolysis of the PGA backbone [25]. Figure 7b illustrates that the degradation efficiency of pure PGA yarn can reach 30% following hydrolysis at 60 °C for 24 h. Nevertheless, the degrading efficiency of the composite yarns, following the incorporation of GO and CNTs, diminished to a certain degree. The SEM results indicate that the incorporation of GO and CNTs results in increased diameters of the nanofibers, consequently reducing the specific surface area of the fibers produced. This diminishes the likelihood of fiber interaction with water molecules, leading to a reduction in hydrolysis efficiency. Analysis of the degradation rates of composite yarns including GO and CNTs reveals that the incorporation of GO markedly diminishes the degradation efficiency of the yarns. This may result from the GO surface possessing a greater number of oxygen-containing functional groups, which, when compounded with PGA, exhibit a stronger interaction with the PGA molecular chain, thereby enhancing the stability of the PGA crystal structure and partially inhibiting the hydrolysis reaction.

## 4. Conclusions

By utilizing recycled PGA as a raw material, it is possible to create yarns that exhibit enhanced mechanical properties through the incorporation of carbon nanomaterials like GO and CNTs, followed by electrospinning into nanofibers and subsequent twisting. The findings indicated that uniform nanofibers can be produced through the electrospinning of recycled PGA in HFIP at a concentration of 15 wt%. The mechanical strength of the resultant PGA can be improved by incorporating an appropriate amount of GO during the electrospinning process. The yarn produced from the PGA/GO nanofiber film containing 3 wt% of GO exhibited an enhanced tensile strength of 21.36 MPa and an elastic modulus of 259.51 MPa. As a comparison, the addition of CNTs in the range of 0–3 wt% resulted in a reduction in the mechanical properties of the PGA/CNT nanocomposite yarns. The degradation efficiency of the resulting nanocomposite yarns in water exhibited a slight decrease following the incorporation of GO or CNTs, with the most pronounced reduction observed upon the addition of 1 wt% of GO. The degradation behavior of the PGA/GO yarns in water over an extended duration should be studied in detail in the future, particularly to elucidate the kinetics of the degradation process and the environmental impact of the intermediate and final products. The resultant PGA/GO yarns exhibit characteristics such as a relatively low cost, good mechanical strength, and a rapid degradation rate. These properties enable their further weaving into textiles, temporary blocking balls, and applications in engineering fields like shale gas exploitation.

## Figures and Tables

**Table 1:** Tensile properties of PGA and PGA-based nanocomposite yarns.

Samples	PGA/Nanofiller (wt/wt)	Tensile Strength (MPa)	Elongation at Break (%)	Elastic Modulus (MPa)
PGA	100/0	20.86 ± 0.57	91.11 ± 7.28	180.19 ± 41.27
PGA/GO	99/1	18.08 ± 1.15	38.75 ± 15.1	246.23 ± 21.95
PGA/GO	97/3	21.36 ± 0.85	60.98 ± 18.47	259.51 ± 29.72
PGA/GO	95/5	8.18 ± 2.84	17.23 ± 9.71	113.09 ± 34.87
PGA/GO	90/10	2.23 ± 1.12	2.31 ± 0.81	120.94 ± 18.53
PGA/CNT	99/1	16.61 ± 1.3	46.06 ± 13.08	151.28 ± 25.97
PGA/CNT	97/3	16.75 ± 1.75	61.19 ± 16.81	103.74 ± 27.58

## Data Availability

The original contributions presented in this study are included in the article/Supplementary Material. Further inquiries can be directed to the corresponding author.

## Supplementary Materials

The following supporting information can be downloaded at: https://www.mdpi.com/article/10.3390/polym17010100/s1. Figure S1: Typical atomic force microscope (AFM) image of GO nanosheets; Figure S2: Typical SEM image of CNT powders used in this work; Figure S3: Tensile strength of PGA yarns made of nanofiber films electrospun under different voltages; Figure S4: SEM images of electrospun PGA/CNT nanocomposite fibers. (a–c) PGA:CNT = 99:1 (wt:wt); (d–f) PGA:CNT = 97:3 (wt:wt); Table S1: Mechanical properties of PGA yarns twisted along the different directions from the same electrospun nanofiber film.
