# Supplementary material for: High-Strength and Rapidly Degradable Nanocomposite Yarns from Recycled Waste Poly(glycolic acid) (PGA)
Source: Polymers (Basel). 2025 Jan 2;17(1):100. doi: 10.3390/polym17010100 (PMC11722973; doi:10.3390/polym17010100)
Supplement: Supplementary file 1 [file polymers-17-00100-s001.zip › polymers-3349368-supplementary.pdf]

Supporting Information

High-Strength and Rapidly Degradable Nanocomposite Yarns from Recycled Waste Poly(glycolic acid) (PGA)

Ben Liu [1,2], Shixiao Wang [3], Hanling Guo [3], Huibo Yin [1,2], Yuqiu Song [3], Min Gong [3], Liang Zhang [3], Xiang Lin [3]
and Dongrui Wang [3,*]

[1] State Key Laboratory of Shale Oil and Gas Enrichment Mechanisms and Effective Development, No. 197 Baisha Road, Shahe Town, Beijing 102206, China
[2] Research and Development Center of Measurement and Control Technology and Equipment, SINOPEC Research Institute of Petroleum Engineering Co., Ltd., No. 197 Baisha Road, Shahe Town, Beijing 102206, China
[3] School of Chemistry and Biological Engineering, University of Science and Technology Beijing, No. 30 Xueyuan Road, Haidian District, Beijing 100083, China
* Correspondence: wangdr@ustb.edu.cn

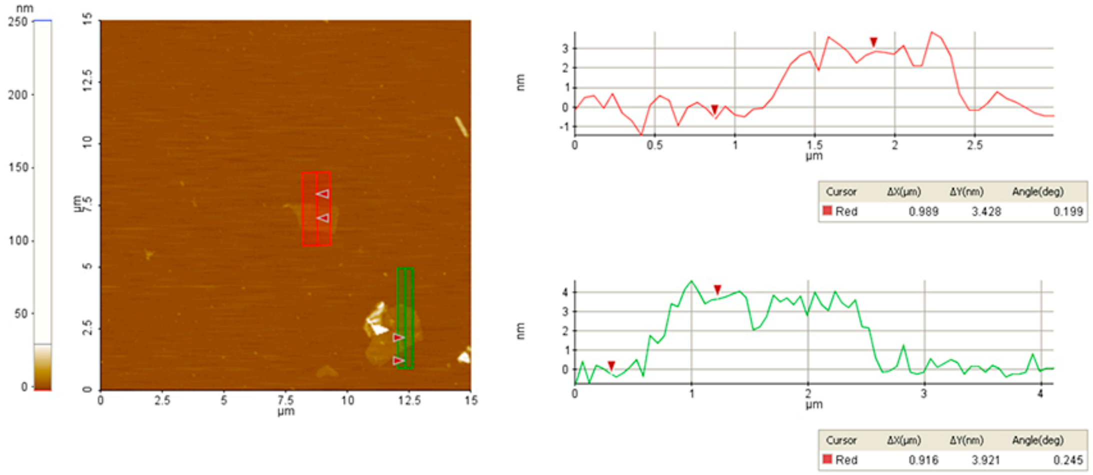

Figure S1. Typical atomic force microscope (AFM) image of GO nanosheets.

The two nanosheets in the image show lateral size of ca. 1 μm and thickness of ca. 3-4 nm.

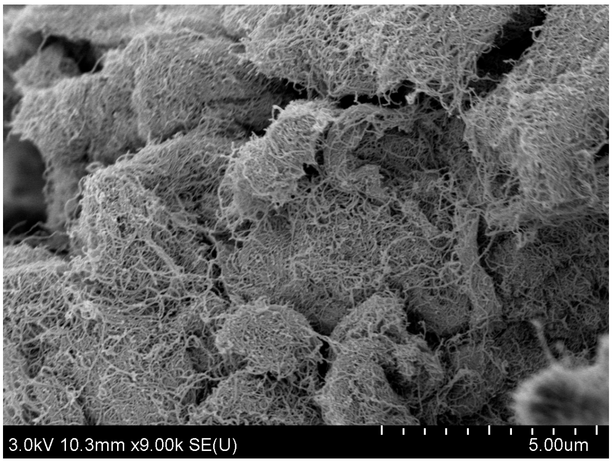

Figure S2. Typical SEM image of CNT powders used in this work.

Table S1. Mechanical properties of PGA yarns twisted along the different directions from the same electrospun nanofiber film.

Samples	Tensile strength (MPa)	Elongation at break (%)	Elastic modulus (MPa)
Parallel direction-1	20.98	87.4	190.97
Parallel direction-2	20.81	108.92	170.33
Parallel direction-3	21.34	100.60	233.53
Parallel direction-4	21.27	96.65	163.63
Parallel direction-5	21.05	78.74	193.55
Perpendicular direction-1	2.12	114.7	38.5
Perpendicular direction-2	2.18	113.4	30.2
Perpendicular direction-3	2.17	129.5	21.88

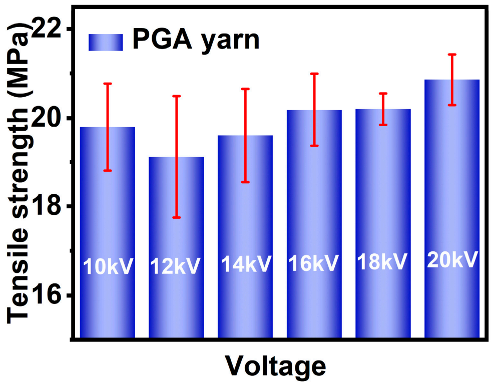

Figure S3. Tensile strength of PGA yarns made of nanofiber films electrospun under different voltages.

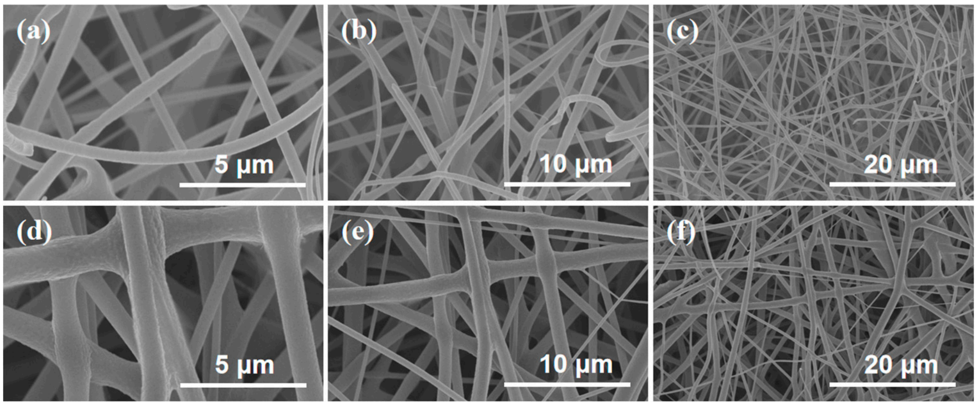

Figure S4. SEM images of electrospun PGA/CNT nanocomposite fibers. (a)-(c) PGA:CNT = 99:1 (wt:wt); (d)-(f) PGA:CNT = 97:3 (wt:wt).
